# Germline *RET* Leu56Met Variant Is Likely Not Causative of Multiple Endocrine Neoplasia Type 2

**DOI:** 10.3389/fendo.2021.764512

**Published:** 2021-12-01

**Authors:** Anna Reimer Hansen, Line Borgwardt, Åse Krogh Rasmussen, Christian Godballe, Morten Møller Poulsen, Filipe G. Vieira, Jes Sloth Mathiesen, Maria Rossing

**Affiliations:** ^1^ Center for Genomic Medicine, Copenhagen University Hospital, Copenhagen, Denmark; ^2^ Department of Endocrinology and Metabolism, Copenhagen University Hospital, Copenhagen, Denmark; ^3^ Department of Otorhinolaryngology, Head & Neck Surgery and Audiology, Odense University Hospital, Odense, Denmark; ^4^ Department of Clinical Research, University of Southern Denmark, Odense, Denmark; ^5^ Department of Endocrinology and Internal Medicine, Aarhus University Hospital, Aarhus, Denmark; ^6^ Department of Clinical Medicine, University of Copenhagen, Copenhagen, Denmark

**Keywords:** multiple endocrine neoplasia type 2, medullary thyroid cancer, RET, Leu56Met, Genetics

## Abstract

Activating variants in the receptor tyrosine kinase *RE*arranged during *T*ransfection (RET) cause multiple endocrine neoplasia type 2 (MEN 2), an autosomal dominantly inherited cancer-susceptibility syndrome. The variant c.166C>A, p.Leu56Met in *RET* was recently reported in two patients with medullary thyroid cancer (MTC). The presence of a pheochromocytoma in one of the patients, suggested a possible pathogenic role of the variant in MEN 2A. Here, we present clinical follow up of a Danish *RET* Leu56Met cohort. Patients were evaluated for signs of MEN 2 according to a set of predefined criteria. None of the seven patients in our cohort exhibited evidence of MEN 2. Furthermore, we found the Leu56Met variant in our in-house diagnostic cohort with an allele frequency of 0.59%, suggesting that it is a common variant in the population. Additionally, none of the patients who harbored the allele were listed in the Danish MTC and MEN 2 registries. In conclusion, our findings do not support a pathogenic role of the Leu56Met variant in MEN 2.

## Introduction

*RE*arranged during *T*ransfection (RET) is a receptor tyrosine kinase that plays essential roles in several intracellular pathways as well as processes such as embryonic development of the enteric nervous system. The RET protein, which is encoded by the *RET* gene on chromosome 10, is composed of three functional domains. These include an intracellular tyrosine kinase domain, a transmembrane domain and an extracellular ligand binding domain, containing cadherin-like domains and a cysteine-rich domain important for receptor dimerization (1). Germline loss-of-function variants in *RET* cause Hirschsprung’s disease (HSCR), a congenital intestinal malformation (2), while activating variants cause multiple endocrine neoplasia type 2 (MEN 2), an autosomal dominantly inherited cancer-susceptibility syndrome (3, 4). The MEN 2A subtype is characterized by medullary thyroid cancer (MTC) with pheochromocytoma, primary hyperparathyroidism (PHPT) and in some cases, cutaneous lichen amyloidosis and HSCR, while MEN2B is characterized by MTC, pheochromocytoma, gastrointestinal and/or mucosal neuromas and a marfanoid habitus (1, 5).

Several recurrent hotspot variants in *RET* have been identified in MEN 2 patients, and there appears to be some genotype-phenotype correlation, allowing for MTC risk stratification and possible early surgical intervention in affected individuals (6). However, for variants identified more sporadically, the causality may not be as clear, resulting in possible misclassification of the variants and subsequent inaccurate patient management (7–10).

The variant c.166C>A, p.Leu56Met in exon 2 of *RET* (NM_020975.5) was initially reported in a patient with HSCR (11) but was recently identified in two patients with MTC (12). The coinciding presence of a pheochromocytoma in one of the patients led the authors to suggest a possible pathogenic role of the Leu56Met variant in MEN 2A. In order to determine if the variant is associated with MEN 2, we present clinical follow up of a Danish *RET* Leu56Met cohort and the allele frequency of the variant from our in-house diagnostic cohort.

## Patients and Methods

Our cohort consisted of patients who underwent genetic testing for MEN 2, MTC, PHPT or pheochromocytoma at the Center for Genomic Medicine, Copenhagen University Hospital, between 2014 and 2019. Carriers of *RET* Leu56Met were selected from the cohort and evaluated for symptoms of MEN 2 by reviewing patient files and by subsequent biochemical follow up at the Department of Endocrinology, Copenhagen University Hospital. An additional patient from Department of Endocrinology, Aarhus University Hospital was included and biochemical follow up was conducted. None of the Leu56Met carriers harbored additional variants in *RET.*

Evidence of MEN 2 in *RET* Leu56Met carriers was evaluated by the following criteria, as previously described (10) with additional criteria to include MEN2B: (i) the patient demonstrates more than one MEN 2 manifestation, including histologically verified MTC, histologically verified pheochromocytoma, histologically verified gastrointestinal or mucosal neuromas, histologically verified HSCR, biochemically verified PHPT, and clinically or histologically verified cutaneous lichen amyloidosis, or (ii) the patient has one MEN 2 manifestation and a relative with MTC and the *RET* Leu56Met variant.

Data was compiled from patients for whom *RET* sequencing was performed at our department until 2020, as part of a larger gene panel for hereditary endocrinological diseases, renal cancers and malignant melanoma. These patients constituted our in-house diagnostic cohort. Data from this cohort was bioinformatically processed to identify any carriers of the Leu56Met variant.

Genetic testing was conducted by next generation sequencing as previously described (13). This study was approved by the local Ethics Committee in the capital region of Denmark (H-4-2010-050) as well as the Danish Health Authority (3‐3013‐395/3) and the Danish Data Protection Agency (18/17801).

## Results

We identified seven unrelated *RET* Leu56Met carriers in our cohort (**Table 1**). None of the carriers exhibited more than one MEN 2A or MEN 2B manifestation or had any family history of MTC. Moreover, none of the patients were diagnosed with HSCR or cutaneous lichen amyloidosis. Consequently, the Leu56Met carriers from our cohort did not fulfill the predefined criteria and were not considered to show evidence of MEN 2.

**Table 1:** *RET* Leu56Met carriers identified on suspicion of hereditary PHPT, MTC or PHEO.

Patient No.	Sex	Age^a^	*RET* test indication	Surgery	Biomarkers MTC	Biomarkers PHEO		Biomarkers PHPT			
					s-calcitonin, basal^b^	p-Metanephrines^c^, time of diagnosis	p-Metanephrines^c^, follow up	p-Calcium ion^d^, time of diagnosis(mmol/L)	p-Calcium ion^d^, follow up 2019(mmol/L)	p-Parathyrin (PTH)^e^, time of diagnosis(pmol/L)	p-Parathyrin (PTH)^e^, follow up 2019(pmol/L)
**1**	F	37/40	PHPT	No	Normal	Normal	–	*1.36*	*1.42*	*8.1*	*9.2*
**2**	F	35/35	PHPT	Yes^f^	–	–	–	*1.56*	1.19	*18.0*	*8.4*
**3**	M	-/66	PHPT	Yes^f^	–	–	–	*1.37*	*1.36*	*20.4*	*13.3*
**4**	M	48/49	PHPT	No	–	–	–	*1.42*	1.32	*15.3*	*8.3*
**5**	F	44/64	PHPT	Yes^f^	–	–	–	1.24	1.32	*9.6*	*9.0*
**6**	F	74/81	PHEO	Yes^g^	Normal	Elevated	Normal	–	1.23	–	*13.3*
**7**	M	74/77	PHEO	Yes^g^	Normal	Elevated	Normal	1.29	1.26	5.5	4.7

a Age at time of diagnosis/age at latest clinical evaluation.

b Reference interval <2,79 pmol/L (males), <1,87 pmol/L (females).

c Reference interval 3-Methoxyadrenalin <90 ng/L/<0,45 nmol/L, 3-Methoxynoradrenalin <180 ng/L/<1,07 nmol/L.

d Reference interval 1.18-1.32 mmol/L. Values in italics are elevated.

e Reference interval 1.6-6.9 pmol/L. Values in italics are elevated.

f Surgery for PHPT.

g Surgery for PHEO.

MTC, medullary thyroid cancer; PHEO, pheochromocytoma; PHPT, primary hyperparathyroidism; -, not available.

We further screened our in-house cohort of diagnostic samples for the Leu56Met variant. In this cohort the variant was found with an allele frequency of 0.59% (36 of 6066 alleles). None of the patients who harbored the allele were found in the Danish MTC cohort (1960–2014) (14) or the Danish MEN 2 cohorts (1901–2021) (15, 16, Unpublished data).

## Discussion

In this study, we present a cohort of Danish carriers of the *RET* Leu56Met variant. None of the carriers display evidence of MEN 2 or MTC according to our predefined criteria. As some of the patients who only presented with PHPT were relatively young, it is possible that they could develop MTC later in life. However, a large study recently reported PHPT as a first manifestation of MEN 2A in less than 1% of index cases (17), rendering this possibility unlikely. Our analysis of the cohort is based on biomarkers to assess the presence of MTC. While non-secretory MTCs have been reported, these are exceedingly rare (18), and have never been identified as part of hereditary MTC in our MEN 2 cohort. Additionally, we find the Leu56Met variant with an allele frequency of 0.59% in our in-house diagnostic cohort. None of the patients who harbored the allele were listed in the Danish MTC and MEN 2 registries. Relatives of the Leu56Met carriers in our cohort were not examined for carrier status, however no family history of MEN 2 was reported. Considering these factors, the Leu56Met variant is most likely a benign variant. Thus, our findings dispute the previously suggested association between the Leu56Met variant and MEN 2A (12).

The *RET* Leu56Met variant was first described in a patient with short segment HSCR, but no MEN 2 manifestations (11). Subsequently, the Leu56Met variant has also been described in a fetus with bilateral renal agenesis (19), a patient with congenital abnormalities of the kidneys and urinary tract also carrying a frameshift variant in *TBC1D1* (20), and in a family with periodontal disease also carrying a variant in *IRF8* (21). Furthermore, the *RET* Leu56Met variant has previously been detected in control subjects. Johnston et al. found the Leu56Met variant in 6 of 571 individuals (1.1%) not selected for a personal or family history of cancer (22). Another large study of a healthy ancestrally diverse cohort identified Leu56Met with an allele frequency of 0.76% in the European subpopulation (23). The frequent findings of the variant in these studies indicate that Leu56Met is a common variant in the general population. Indeed, it has been reported in gnomAD with an allele frequency of 0.48% in the non-Finnish European population, including one individual homozygous for the variant^1^. In comparison, two of the known pathogenic variants causative of MEN 2, p.Cys634Tyr and p.Met918Thr (6) are both reported in gnomAD with allele frequencies below 0.001%. Even the possibly low penetrant pathogenic variant p.Val804Met (24) is reported with an allele frequency of 0.014% in gnomAD. The relatively high allele frequency of the Leu56Met variant in our in-house diagnostic cohort suggests that it is a common variant without clinical significance in the Danish population.

Leu56 is located in exon 2 of *RET*, while the pathogenic variants that give rise to MEN 2 are primarily reported in exons 5, 8, 10, 11, 13, 14, 15, and 16 (5). RET is comprised of three functional domains, and Leu56 is positioned within the extracellular cadherin-like domain, where loss-of-function variants were originally identified in HSCR patients (25). Mutation of this residue could potentially result in loss of function by misfolding of the protein and subsequent degradation in the endoplasmic reticulum, leading to a lack of secretion to the cell surface. However, RET protein containing the Leu56Met variant was shown to have a secretion efficiency similar to that of wildtype RET (26). Additionally, RET Leu56Met showed no significant difference in RET phosphorylation, or phosphorylation of downstream targets ERK and STAT3, compared to wildtype protein. In fact, the Leu56Met variant was applied as a negative or nonpathogenic control in these functional studies (27, 28). Furthermore, *in silico* analyses overall do not support a damaging effect of the amino acid substitution, although there is some discrepancy between the various tools (REVEL: 0.5, range from 0-1 where higher values predict pathogenicity; CADD: 8.7, range from 1-99 where higher values predict pathogenicity; BLOSUM62 score: 2, positive score indicates that the specific amino acid alteration is more likely to occur; Grantham distance: 15, range from 5-215 where higher values predict pathogenicity) (29–32).

A diagnosis of MEN 2 entails major individual implications with regards to treatment and follow-up, including the psychological aspects of a lifelong risk of developing MEN 2 related manifestations (5, 33, 34). Thus, it is important to correctly distinguish between pathogenic variants in *RET* and benign polymorphisms found in the population. Objectively, it is possible that Leu56Met represents a frequent pathogenic variant with very low penetrance, thus conveying a low lifetime risk of developing MTC, as recently reported for the pathogenic *RET* variant p.Val804Met (24). However, most reported pathogenic *RET* variants are highly penetrant and confer a moderate to high lifetime risk of MTC (6). The Leu56Met variant could also, although not inherently pathogenic, potentially act as a genetic modifier and increase the risk of other cancers. The male patient 1 described by Paragliola et al. was diagnosed with both MTC and pheochromocytoma, and thus MEN 2A cannot be ruled out. However, the diagnosis of pheochromocytoma in a patient without abnormal levels of metanephrines and catecholamines is highly unusual. Taken together, the results from our study and larger population studies (23, 35), suggests that the identification of the *RET* Leu56Met variant in the patient reported in Paragliola et al. most likely reflects the high allele frequency in the general population, rather than a pathogenic finding causative of MEN 2.

Based on results from our cohorts, we found no evidence to support a causative role of the *RET* Leu56Met variant in MEN 2. Consequently, we suggest that Leu56Met is most likely a benign variant.

## Data Availability Statement

The original contributions presented in the study are included in the article/supplementary material. Further inquiries can be directed to the corresponding author.

## Ethics Statement

The studies involving human participants were reviewed and approved by Ethics Committee in the capital region of Denmark (H-4-2010-050), Danish Health Authority (3‐3013‐395/3), Danish Data Protection Agency (18/17801). Written informed consent for participation was not required for this study in accordance with the national legislation and the institutional requirements.

## Author Contributions

AH drafted the manuscript, coordinated the study and carried out the data analysis. MR, LB and JM conceived the study, facilitated the collaboration and participated in the drafting of the manuscript. FV carried out the bioinfomatic processing and participated in drafting the manuscript. ÅR and MP participated in data collection and drafting of the manuscript. CG participated in review of patient cohorts and drafting of the manuscript. All authors contributed to the article and approved the submitted version.

## Conflict of Interest

The authors declare that the research was conducted in the absence of any commercial or financial relationships that could be construed as a potential conflict of interest.

## Publisher’s Note

All claims expressed in this article are solely those of the authors and do not necessarily represent those of their affiliated organizations, or those of the publisher, the editors and the reviewers. Any product that may be evaluated in this article, or claim that may be made by its manufacturer, is not guaranteed or endorsed by the publisher.

## Footnotes

1
https://gnomad.broadinstitute.org/, accessed 11^th^ August 2021
